# On the Progress of Galvanism

**Published:** 1801-09

**Authors:** 


### *On the Progress of Galvanism.*

OUR Readers have already had their attention turned to the curious and interefting fubject of Galvanic Electricity.* Since the period of thofe communications, the fubject has been profecuted with great induftry and ability at the Royal Inftitution; and we have reafon to believe, that all the improvements or difcoveries of importance, fince that time, claimed by our neighbours, have been made in this country.

" When the great intenfity of the fhock is confidered, and at the fame time the minutenefs of the fpark, and weak appearances of other electrical phenomena, the neceffity of the hands being wetted to receive the fhock, its not breaking, though fo very ftrong, through any confiderable fpace of air, nor occafioning any or fcarcely any report, as well as from the different kind of fenfation it produces, it is not furprizing that feveral philofophers fhould doubt its being an electric phenomenon, or if it be, that it is much modified by unknown circumftances. We ought to confider, however, that it is the character of electric fhocks, which are felt to equal diftances from the extremities of an animal, that thofe which are produced by a fmall quantity of electricity at a high intenfity, caufe a more fudden and tranfient fenfation than thofe produced by a much larger quantity at a low intenfity, and therefore probably moving more flowly. When a quantity of electricity, for inftance that produced by fix turns of a machine, is put into a fmall Leyden phial, a fmart quick fhock will be felt, accompanied with a loud report, and the electric fluid will move through a confiderable fpace of air, and fhew a very lucid fpark. The electrometer will likewife indicate a high intenfity before the difcharge. But if the fame quantity be put into a phial having four times the coated furface, the electrometer will only fhew $\frac{1}{4}$ of the intenfity: the fpark will break through only $\frac{1}{16}$ part of the fpace that it did in the former inftance, accompanied with a very feeble fpark; but the fhock will be the fame to a perfon who receives it through his body, becaufe the fame quantity of electric fluid muft pafs through him, though it is eafy to conceive that the fenfation will be different, as it probably moves with lefs velocity in the laft cafe than in the firft. If, inftead of a phial, we fuppofe the electricity to be difperfed over the furface of a large

* Journal, Vol. I. p. 53, and Vol. IV. p. 115.

battery, the intenſity diſcoverable by the electrometer will be very ſmall indeed: the ſtriking diſtance will be almoſt nothing, and the ſpark much diminiſhed, though the ſhock muſt ſtill be equally powerful. It would appear from ſome ingenious calculations made by Mr. Nicholſon, that the celerity with which the Galvanic pile produces electricity, is two hundred times more rapid than what can be produced by the labour of one man with a powerful electrical machine, while at the ſame time its intenſity is ſo low, that the ſhock may be conſidered as produced much more by the maſs, or quantity of electricity, than by the velocity of its motion.

"Some of the moſt curious properties of this apparatus of Signor Volta ſtill remain to be mentioned: we mean its action as a chemical agent, in which its effects are much more powerful than common electric machines, and uncommonly intereſting. When the Galvanic influence is directed in a proper manner through water, that fluid is decompoſed, and ſeparated into its component parts, *viz.* oxygen and hydrogen, which appear in the form of gas, and in their proper proportions. This curious fact was firſt noticed by Mr. Carliſle and Mr. Nicholſon. The circumſtances which led to this diſcovery are deſcribed by the latter gentleman in his Journal, Vol. IV. p. 182, which may be conſulted by the reader. We ſhall content ourſelves here with pointing out the method of making the experiment. If a glaſs tube, about ſix inches long, and half an inch in diameter, be filled with diſtilled water, which is confined in it by means of two corks, and if two copper or iron wires, communicating with the top and bottom of the pile, be made to paſs through the corks, ſo as to be about two or three inches diſtant from each other; if the loweſt piece of the pile be ſilver, a fine ſtream of gas, in very minute bubbles, will immediately iſſue from the loweſt wire, and riſe to the top, where they will collect: this wire will remain very clean and bright. From the upper wire, which is connected with the zinc end of the pile, no gas is obſerved to riſe; but the wire itſelf oxidates very quickly, and the oxid gradually ſubſides in the form of very fine clouds: during this operation the water is diminiſhed in quantity. If this gas be examined in the uſual manner, it is found to be very nearly pure hydrogen. In this experiment it would appear that the hydrogen is ſeparated from the water, and converted into a gaſeous ſtate, by the wire connected with the ſilver end of the pile, while the oxygen unites itſelf with the wire connected with the zinc end. When the pile is inverted, the hydrogen gas ariſes from the upper wire, and the lower oxidates. That this effect is produced by the Galvanic influence is evident; for on making a communication, by means of a conductor, between the upper upper and lower extremities of the pile, the emiſſion of gas is ſuſpended, but proceeds again, as ſoon as the communication is removed.

"If two unoxidable wires, as for inſtance wires of gold or platina, be uſed, then a ſtream of gas iſſues from each, and the water is diminiſhed. This gas is found to be a mixture of hydrogen and oxygen, and explodes violently on the approach of an ignited body.

"In order to procure theſe two gaſes ſeparate from each other, to eſtimate their quantities, and examine their nature more particularly, Mr. Cruikſhank took a glaſs tube, ten inches in length, and by means of a blow-pipe bent it in the middle until the legs formed an acute angle, reſembling the letter V. While the glaſs was red-hot, he contrived to blow an opening at the angle, about one-tenth of an inch, or a little more, in diameter. Two gold wires, paſſed through corks ſecured by cement, were introduced into the legs, and brought within an inch of each other at the bend: the tube was then filled with diſtilled water, and a finger placed on the opening at the angle, to prevent the fluid from eſcaping. It was placed in a tea-cup containing water, with the angle downwards, the legs having an inclination of about forty-five degrees. The extremities of the wires being then brought into contact with thoſe of the pile, a quantity of gas was diſengaged from both; but by far the moſt from that connected with the ſilver. By this contrivance, the gaſes from the two wires were obtained perfectly diſtinct, each gas aſcending in the leg of the tube which contained its generating wire. On examining the gaſes, that from the wire connected with the ſilver end of the pile was almoſt pure hydrogen, and that from the zinc end almoſt pure oxygen, in the proportion of two parts by meaſure of the former to one of the latter, or nearly in the proportion in which they exiſt in water.

"When the glaſs tube is filled with diſtilled water, to which a little tincture of litmus is added, and a communication made by gold wires, a quantity of gas ariſes from both wires; but in greateſt quantity from that connected with the ſilver. In a few minutes a fine red line is ſeen, extending upwards from the extremity of the zinc wire *, and in a ſhort time the whole fluid near the point of this wire becomes red: the fluid, however, about the ſilver wire looked of a deeper blue than before, the ſlight tinge of purple being deſtroyed.

* For the ſake of brevity, the wire connected with the zinc end of the pile is called the zinc wire, and that connected with the ſilver end, the ſilver wire, as was done by Mr. Cruikſhank.

" If the tube be filled with diftilled water, tinged with tincture of Brazil-wood, the fluid furrounding the filver wire, particularly towards its extremity, becomes purple; and this tinge increafes fo faft, that the whole fluid furrounding this wire foon affumes as deep a colour as can be produced from ammonia, while the portion of the fluid in contact with the zinc wire becomes very pale, and almoft colourlefs.

" Thefe experiments would feem to fhew that an acid, probably the nitrous, is produced at the wire proceeding from the zinc, which may arife from the union of the oxygen of the decompofed water with the azote of the common air diffolved in the water: at the fame time, an alkali, probably ammonia, appears to be formed at the filver wire, moft likely from the union of the nafcent hydrogen of the decompofed water with the azote of the atmofpheric air.

" In reflecting on thefe experiments, it appears, that in fome of them the water is decompofed; but how this is effected is by no means eafy to be explained. For example, it feems extremely myfterious how the oxygen fhould pafs filently from the extremity of the filver wire to that of the zinc wire, and there make its appearance in the form of gas. It is to be obferved, likewife, that this effect takes place whichever way the wires are placed. The theory offered to the public by Mr. Cruikfhank is certainly ingenious. He thinks that the fimpleft and eafieft mode of explanation would be to fuppofe, that the Galvanic influence (whatever it may be) is capable of exifting in two ftates, that is, in an oxygenated and deoxygenated ftate. That when it paffes from metals to fluids containing oxygen, it feizes their oxygen, and becomes oxygenated; but, when it paffes from the fluid to the metal again, it affumes its former ftate, and becomes de-oxygenated. Now, when water is the fluid interpofed, and the influence enters it from the filver fide, de-oxygenated, (and he fuppofes that it always paffes from the de-oxygenated to the oxygenated fide), it feizes the oxygen of the water, and difengages the hydrogen, which accordingly appears in the form of gas; but, when the influence enters the zinc wire, it parts with the oxygen with which it had formerly united, and this either efcapes in the form of gas, or unites with the metal to form an oxyd, or combined with fome of the azote contained in the water may form nitrous acid.

" It is a well known chemical fact, that hydrogen gas, in its nafcent ftate, reduces the oxyds of metals; it was natural to expect, therefore, that by filling the glafs tube with a metallic folution, the hydrogen would feparate the oxygen from the metallic oxyd, and procure the metal in its pure ftate. Accordingly, when the tube is filled with a folution of acetite of lead in in diftilled water, and a communication made in the ufual way, no gas is perceived to iffue from the filver wire, but in a few minutes beautiful metallic needles are perceived on the extremity of this wire: thefe foon increafe, and affume the form of a fern, or other vegetable. The lead thus feparated is perfectly in its metallic ftate, very brilliant.

"When a folution of fulphat of copper is employed, the copper is precipitated in its metallic ftate, but inftead of appearing in cryftals, it forms a kind of button, which adheres firmly to the end of the wire.

"On making the experiment with a folution of nitrat of filver, the filver is precipitated in the form of a beautiful metallic brufh, the metal fhooting into fine needle-like cryftals.

"Thefe phenomena may be explained on Mr. Cruickfhank's hypothefis, by fuppofing the Galvanic influence, in paffing from the filver wire, feizes the oxygen of the metallic oxyde, and afterwards depofits it on entering the zinc wire. In this cafe, no gas fhould appear at the filver wire; but, when a perfect metal is employed, oxygen fhould be difengaged from the zinc one; and this is exactly what takes place. What Mr. Cruickfhank confiders, however, as the ftrongeft argument in favour of this hypothefis, is, that all fluids which do not contain oxygen, are incapable of tranfmitting the Galvanic fluid; fuch as alcohol, ether, fat, and the effential oils, as he has proved by direct experiments: but, on the contrary, that all thofe which do contain oxygen, conduct it more or lefs readily, as all aqueous fluids, metallic folutions, and acids, more efpecially the concentrated fulphuric acid, which it decompofes. By this theory, alfo, we can eafily explain the oxydation of the zinc plates in the machine, where the Galvanic fluid, in paffing through the different plates, appears to be alternately oxygenated and de-oxygenated.

"Having found that metallic folutions were decompofed by the Galvanic influence, Mr. Cruickfhank wifhed to afcertain if their folution in alkalies, and more particularly ammonia, could be decompofed in the fame way. For this purpofe, to a dilute folution of the nitrat of filver he added fome pure ammonia, until the mixture fmelled ftrongly of the latter fubftance. The mixture was introduced into the glafs tube, and filver wires applied. When the tube was placed in the circle of communication, a very rapid production of gas took place from the wire connected with the filver extremity of the pile, although little or none efcaped from the zinc wire during the procefs.

"After fome time a quantity of greyifh flakes, evidently metallic filver, were feparated by the wire which gave out the gas, and a dark grey powder was feparated from the zinc wire.

After

After some hours, the apparatus was removed : at this time a considerable quantity of metallic silver was deposited, and the zinc wire was encrusted by a blueish-black substance. On endeavouring to remove this crust with the finger, part of it exploded, though still moist: the wire was found to be very much corroded and full of holes. Next morning, the powder which adhered to the wire, being dry, was touched with a knife, when it exploded with a very considerable noise. There can be little doubt, that these explosions proceeded from the fulminating silver of Berthollet, which must have been formed at the zinc wire. The experiment was repeated with pure ammonia, instead of the solution of silver, which answered almost equally well; for the silver of the zinc wire, after being corroded, was taken up by the ammonia, and afterwards deposited, in its metallic form, by the silver wire. A small quantity of fulminating silver was likewise formed.

“ A quantity of pure ammonia being put into the tube, and platina wires being introduced as conductors, a rapid production of gas took place from both wires, which, on being examined, was found to consist of hydrogen and azotic gas, very nearly in the same proportion as they exist, according to Berthollet, in ammonia. Here, undoubtedly, the ammonia was decomposed by the Galvanic influence. The oxygen disengaged at the zinc wire uniting with the hydrogen, while the azote of the ammonia escaped in the form of gas, and became mixed with the hydrogen, disengaged at the same time from the silver wire.

“ From these experiments it will be perceived, that the Galvanic influence is a very powerful chemical agent, and that, by means of it, decompositions may be made much more quickly, and with infinitely less trouble, than by an electrical machine. It might, indeed, probably be employed with success in the analysis of minerals, more particularly in separating lead, copper, and silver, from their different solutions.

“ Since the above account was drawn up, some very interesting facts have been published by Mr. Davy, superintendant of the Pneumatic Institution at Bristol, of which, as they throw considerable light on the phenomena of Galvanism, we shall give an abstract.

“ From the experiments made by Mr. Davy, he has been led to the following conclusions:

“ 1. That the zinc in the plates of a Galvanic pile undergoes no oxydation at common temperatures, so long as the water in contact with it is *pure*, or holds in solution no oxygen gas, no nitrous gas, and no acids. A pile, in which the cloths were moistened with distilled water which had been boiled, being introduced troduced into a veſſel of water, and the acceſs of atmoſpheric air cut off, on being examined after two days, the pieces of zinc were found unoxydated: the zinc, though in contact with pure water, was not oxydated, when the pile was placed in hydrogen gas, or *in vacuo*.

" 2. The oxydation of the zinc plates of the Galvanic pile takes place whenever the water in contact with them holds atmoſpheric air, oxygen, nitrous gas, nitrous acid, muriatic acid, &c. in ſolution. Mr. Davy found, likewiſe, that the oxydation took place much more rapidly in pure oxygen gas than in the atmoſphere.

" 3. When the zinc in contact with water, holding in ſolution ſubſtances containing looſe oxygen, or acids, becomes oxydated, theſe ſubſtances become changed, and exert ſome chemical affinities.

" 4. The Galvanic pile ſeems incapable of acting when the water between the pairs of plates is pure, or contains no oxygen, or ſubſtances containing oxygen.

" 5. The pile acts when the water between the plates holds in ſolution atmoſpheric air, oxygen, nitrous gas, nitrous acid, or muriatic acid.

" 6. The power of action of the pile appears to be proportional to the power poſſeſſed by the conducting fluid ſubſtance between the plates to oxydate the zinc. This appears from the zinc oxydating leſs rapidly in nitrous gas than in atmoſpheric air, and leſs rapidly in atmoſpheric air than in oxygen.

" From theſe facts it appears, the pile of Volta acts only when the conducting ſubſtance between the plates is capable of oxydating the zinc, and that, in proportion as a greater quantity of oxygen enters into combination with the zinc in a given time, ſo in proportion is the power of the pile to decompoſe water, and to give the ſhock greater; and it ſeems not unreaſonable to conclude, that the oxydation of the zinc in the pile, and the chemical changes connected with it, are ſome how the cauſe of the electrical effects produced by it.

" Aſſuming the truth of this concluſion, it was eaſy to conceive, that a pile, much more powerful than any hitherto conſtructed, might be made, particularly on the ſuppoſition that the decompoſition of the interpoſed water was not eſſential to the proceſs. Plates of zinc and ſilver, 1,2 inches ſquare, were faſtened in pairs by reſinous cement: eighteen of theſe pairs were connected with each other by cement, and ſo incloſed by it, as to leave water-tight partitions open at one ſide only, between each pair of plates. When muriatic acid was poured between the partitions of this machine, the plates being perpendicular to the horizon, it acted very powerfully: its capability of of decompofing water, and giving the fhock, being at leaft equal to that of a common pile of feventy plates. Diluted nitrous acid made it act ftill more powerfully. When the partitions were filled with water, its action was barely perceptible. Concentrated nitrous acid was poured into them: the fhock was fo powerful as to benumb Mr. Davy's fingers for fome feconds, and he did not dare to take another. In a fecond experiment with ftrong nitrous acid, he ufed only five pairs of plates, and the fhock was full as powerful as from a common pile of thirty plates. Three pairs of plates, with nitrous acid, gave a very fenfible fhock.

"We have now given as diftinct an account as was confiftent with the room alloted for this article, of the phenomena of Galvanifm. The difcovery of Volta, by which its force may be fo greatly augmented, may be reckoned among the moft important with which philofophy has lately been enriched; and there can be little doubt, from the ardour with which experiments are now making, that great light will be thrown by it both on electricity and chemiftry. May we not likewife venture to predict, that the prefent machines, though very ftrong, will, in procefs of time, give way to others, both vaftly more powerful and convenient? Many perfons remember the time when the beft electrical machine was a tube of glafs, which was rubbed by the hand with a piece of leather. The celebrated Franklin ufed a machine of this kind, with which he made experiments before his felect friends. How defective was this, both in power and convenience, when compared with the common machines in ufe; and is it by any means improbable, that the pile of Volta, which is now viewed with fo much aftonifhment, may in a fhort time give way to improvements equally great? The energy of Galvanifm, as a medical agent, remains ftill to be inveftigated; but when we confider the effects which a very fmall quantity of this influence produces on the mufcles of an animal, even after it is deprived of life, it is not, we think, unreafonable to predict, that the powerful current fent for a confiderable time through a difeafed part, may be productive of the beft effects.

"Anatomifts have difcovered, that the electric organs of the torpedo and electrical eel confift of numerous membranous columns, filled with a great number of plates, or pellicles, in the form of thin difcs, feparated from each other by very fmall intervals, which are filled with a kind of moifture or fluid. This organ being formed of conducting fubftances, can neither be referred to the electrophorus, the condenfer, or the Leyden phial, nor to any other fpecies of electrical machine, excitable by friction, or any of the means by which infulated bodies are electrified. The organs of the fifh very much refemble the pile of

Volta

Volta in their conſtruction and their effects: it is compoſed of conductors, is capable of acting in water, and will give inceſſant or re-iterated ſhocks like them; in ſhort, it may be looked upon as an artificial torpedo.

"The electrical eel and torpedo will give a ſhock to a perſon dipping his hands in the water in which they ſwim, though this ſhock is much more feeble than thoſe experienced when theſe fiſhes are graſped in the hands out of water. In the ſame manner, if a wire coming from the bottom of the pile be inſerted into a baſon of water, and another from the top terminate in the ſame baſon, at ſome diſtance from the firſt, on immerſing the hand in the water a ſhock will be perceived, though not ſo ſtrong as when the water does not intervene. This can only be explained on the ſuppoſition that the living body is a better conductor of this influence than water; the hand therefore offers an eaſier and freer paſſage to it than water; part of it is conveyed through it, though part will ſtill paſs through the water, which will diminiſh the ſtrength of the ſhock.

"It is probable, that when the torpedo wiſhes to give a ſhock to an animal which is in contact with it, or which has any part immerſed in the water in which it ſwims, it need only bring into contact ſome parts of the electric organ, which may be ſeparated by ſome ſlight interval. In the ſame way, if the ſmalleſt interruption takes place in the parts of either of the wires, in the experiment above deſcribed, no ſhock will be felt on putting the hand into the baſon; but the inſtant the contact is made complete, the ſenſation will be experienced.

"A very excellent account of Galvaniſm, previous to the diſcovery of Volta's pile, may be found in the Supplement to the Encylopædia Britannica; alſo in Fowler's Eſſay on Animal Electricity.—Valli's Experiments on Animal Electricity.—A paper by Dr. Wells, in the Philoſophical Tranſactions for 1795.—A Diſſertation by Dr. Chriſtopher Heinrick Pfaff, *de Electricitate Animali*, publiſhed at Stutgardt, 1793.—Fabroni on the Chemical action of metals upon each other at the common temperature of the atmoſphere, and upon the explanation of certain Galvanic phenomena, Journal de Phyſique, Tom. VI.

"For an account of Volta's paper, ſee Philoſophical Tranſactions, 1800, part 2d.; alſo Nicholſon's Journal, vol. vi. p. 179, 187, 223, 241, 254, 275, 313, 326, 337, 388, 394, 472."

